# Cyp2C19*2 Polymorphism Related to Clopidogrel Resistance in Patients With Coronary Heart Disease, Especially in the Asian Population: A Systematic Review and Meta-Analysis

**DOI:** 10.3389/fgene.2020.576046

**Published:** 2020-12-22

**Authors:** Ying Sun, Qing Lu, Xuefei Tao, Biao Cheng, Guoxing Yang

**Affiliations:** ^1^Department of Geriatric Cardiology, Sichuan Provincial People's Hospital, University of Electronic Science and Technology of China, Chengdu, China; ^2^Department of Operations Management, Sichuan Provincial People's Hospital, University of Electronic Science and Technology of China, Chengdu, China

**Keywords:** *Cyp2C19*, polymorphism, clopidogrel resistance, meta-analysis, coronary heart disease

## Abstract

In recent years, the relationship between Cyp2C19*2 gene polymorphism and clopidogrel resistance reflected by platelet function assay has been studied extensively, but there is no clear conclusion yet. In order to evaluate the relationship between Cyp2C19*2 gene polymorphism and clopidogrel resistance more accurately, meta-analysis was conducted in this study. The I^2^ value taking 50% as the limit, the heterogeneity is judged as high or low, and then a random effect model or a fixed effect model is selected for statistical analysis. PubMed, EMBASE, Web of Science, CNKI, and China Wanfang database were searched, and the related literatures from the establishment of the database to May 2020 were collected and analyzed by STATA 15.0 software. A total of 3,073 patients were involved in 12 studies, including 1,174 patients with clopidogrel resistance and 1,899 patients with non-clopidogrel resistance. The results of this study showed that allele model (A vs. G): OR = 2.42 (95%CI: 1.97–2.98); dominant model (AA+GA vs. GG): OR = 2.74 (95%CI: 2.09–3.59); recessive model (AA vs. GA+GG): OR = 4.07 (95%CI: 3.06–5.41); homozygous model (AA vs. GG): OR = 5.70 (95%CI: 4.22–7.71); heterozygote model (GA vs. GG): OR = 2.32 (95%CI: 1.76–3.07), the differences were statistically significant. Also, the analysis of the Ethnicity subgroup indicated that the Asian allele model and the other four gene models were statistically significant. In conclusion, Cyp2C19*2 gene polymorphism is strongly associated with clopidogrel resistance. Allele A, genotype GA, AA, and GG + GA can increase clopidogrel resistance, especially in the Asian population.

## Introduction

Coronary Atherosclerotic Herat Disease (CAHD), hereinafter referred to as Coronary Heart Disease (CHD), is a kind of heart disease caused by coronary atherosclerosis, which could induce vascular stenosis or obstruction, coronary circulation disturbance, myocardial ischemia, hypoxia and even necrosis. With the improvement of living standards, the incidence of coronary heart disease is increasing year by year. CHD has become one of the common diseases seriously affecting human health (Brown et al., 2016). At present, Percutaneous Coronary Intervention (PCI) is the main method for the treatment of CHD, but post-operative patients may develop stent thrombosis (ST), which is a stubborn problem of PCI. Some studies have indicated that 12 months after PCI, the incidence of ST is about 1.5% (Harnek et al., 2019). Antiplatelet therapy is an important treatment for CHD to reduce ST after PCI. Dual antiplatelet therapy with clopidogrel and aspirin is the standard therapy for PCI, which can greatly reduce the incidence of subacute thrombosis after PCI (Siasos et al., 2015). The mechanism of thrombosis is so complex that thrombotic events still occur in many post-PCI patients who receive clopidogrel combined with aspirin. As the methods of evaluating platelet function and the definition of antiplatelet drug resistance are different in different studies, the incidence of antiplatelet drug response variability also varies from study to study. Currently, most studies define clopidogrel resistance (CR) as a <10% ADP-induced decrease in the maximum platelet aggregation from the baseline level (Patel et al., 2019). There are many factors affecting the antiplatelet effect of clopidogrel, including age, diabetes, smoking and proton pump inhibitors (Nakagawa et al., 2016; Desai et al., 2017). Moreover, many studies suggest that the polymorphism of genes encoding related functional proteins is also an important factor leading to clopidogrel response variability. The different genotypes lead to different clopidogrel reactivity, hence resulting in different clinical events (Hokimoto et al., 2014; Xiao et al., 2017; Wu et al., 2018). Furthermore, the research on the gene polymorphism of clopidogrel response variability mainly focuses on the coding genes of related functional proteins in absorption, metabolic transformation and binding to the *P2Y12* receptor. Among the genes researched, the study of gene polymorphisms affecting the metabolic transformation of clopidogrel has attracted the most attention. The relationship between *CYP2C19* gene polymorphism and clopidogrel response variability is consistent in different studies, which may suggest of the important role of *CYP2C19* in the two-step metabolic transformation of clopidogrel. The metabolic transformation of clopidogrel in the liver mainly goes through two cytochrome P.450 (CYP)-dependent steps: the first step produces 2.OXO. Clopidogrel is catalyzed by cytochrome *Cyp2C19, CYPlA2*, and *CYP286* in different proportions, and the second step produces active metabolites catalyzed by cytochrome *CYP3A4/5, CYP286, Cyp2C19*, and *CYP2C9* (Ford, 2016). Gene polymorphism amongst different individuals would vary in changes at the level of functional proteins, which thus influence the degree of active metabolites of clopidogrel, and eventually lead to differences in clopidogrel reactions. The variation in clopidogrel response caused by altering Cyp2C19*2 site and ^*^3 site is the most concerned. It is now agreed that clopidogrel resistance occurs when drugs fail to achieve their desired pharmacological effects, which can be analyzed in the laboratory through a variety of platelet functions. So far, some meta-analyses have been conducted to determine the relationship between Cyp2C19*2 gene polymorphism and clinical outcomes, such as thrombosis or stroke (Jin et al., 2011; Pan et al., 2017). However, the researches on the relationship between Cyp2C19*2 gene polymorphism and clopidogrel resistance reflected by platelet function measurement are ongoing, and there is no clear conclusion. Most studies tend to believe that the variant of Cyp2C19*2, allele G → A, could increase clopidogrel resistance (Chen et al., 2010; Cuisset et al., 2011), but some new studies suggest that the variant of allele G → A has nothing to do with clopidogrel resistance (Amin et al., 2017). Therefore, in this study, a meta-analysis was conducted to evaluate the association between clopidogrel resistance and Cyp2C19*2 polymorphism in patients with coronary heart disease.

## Materials and Methods

### Literature Search

We performed this meta-analysis based on Preferred Reporting Items for Systematic Review and Meta-Analysis (PRISMA) (Moher et al., 2009) (Supplementary Table 1). The databases of PubMed, Excerpt Medica Database (EMBASE), Web of Science, National Knowledge Infrastructure (CNKI), China Wanfang were searched to collect relevant literature up to May 2020. The retrieval strategy is as follows: (“cytochrome P450 2C19” OR “*Cyp2C19*”) AND (“genetic polymorphism” OR “allele” OR “genotype” OR “polymorphism”) AND clopidogrel AND (“resistance” OR “platelet reactivity” OR “platelet response”). For studies with overlapping data or the same population, only the most recent group of subjects were included. There are no language restrictions. Literature retrieval was carried out independently, cross-checked by two researchers in each database. If there were differences, they could be resolved through discussion, or decided by the third researcher.

### Inclusion and Exclusion Criteria

#### Inclusion Criteria

(1) The relationship between Cyp2C19*2 gene polymorphism and clopidogrel resistance; (2) The study was a cohort study or a case-control study; (3) Patients were diagnosed with coronary heart disease; (4) All patients received clopidogrel antiplatelet therapy; (5) The study should provide the number of patients with clopidogrel resistance and non-resistance of each genotype; (6) Diagnostic criteria of clopidogrel resistance: percentage of adenosine diphosphate inhibition ≤ 10%, MPA induced by adenosine diphosphate ≥ 50%, angiotensin converting enzyme index ≥ 50%, PRI ≤ 50%, PRU > 208, IPA < 30%.

#### Exclusion Criteria

(1) Studies belong to reviews, letters, or case reports; (2) Studies involve other definitions of clopidogrel resistance; (3) Repeatedly published data from the same study; (4) Non-coronary heart disease studies, such as ischemic stroke; (5) The control group does not meet the Hardy-Weinberg equilibrium (HWE); (6) NOS(Newcastle-Ottawa Scale) score is <6.

### Data Extraction

The information was extracted as follows: first author, publication years, age, country, diagnostic criteria of clopidogrel resistance, clopidogrel load, genotype, and number of patients with clopidogrel resistance. The data were extracted independently by two researchers and discussed and solved by the third researcher when there was disagreement. When the included research information was insufficient, contacting author if possible.

### Literature Quality Evaluation

After reading the literature carefully, the quality of the literature was evaluated according to The NOS (Stang, 2010). The literature with <6 stars was of low quality, and those with 6 stars or more were high quality literature. Only those with an evaluation of 6 stars and above were included in this study.

### Statistical Analysis

Meta-analysis was performed with Stata 15.0 statistical software. *Q*-test was used to test the heterogeneity of the research results. If I^2^ ≥ 50%, or *P* ≤ 0.05 were considered heterogeneity, Random-effects model (REM) was used (Welton et al., 2007). If I^2^ < 50% and *P* > 0.05 were considered no heterogeneity, fixed-effects model (FEM) should be used for data merging (Leonard and Duffy, 2010). The significance of OR value was performed by Z test. We also made a subgroup analysis of Ethnicity, the year of publication, and the definition of clopidogrel resistance. This Meta-analysis included the evaluation of publication bias, using the funnel plot to determine whether it was symmetrical or not. If the funnel chart was asymmetric, publication bias may exist. Egger's Test was used to test the publication bias. Finally, sensitivity analysis was performed for the robust of results.

## Results

### Basic Information of Research Data

**Figure 1:**
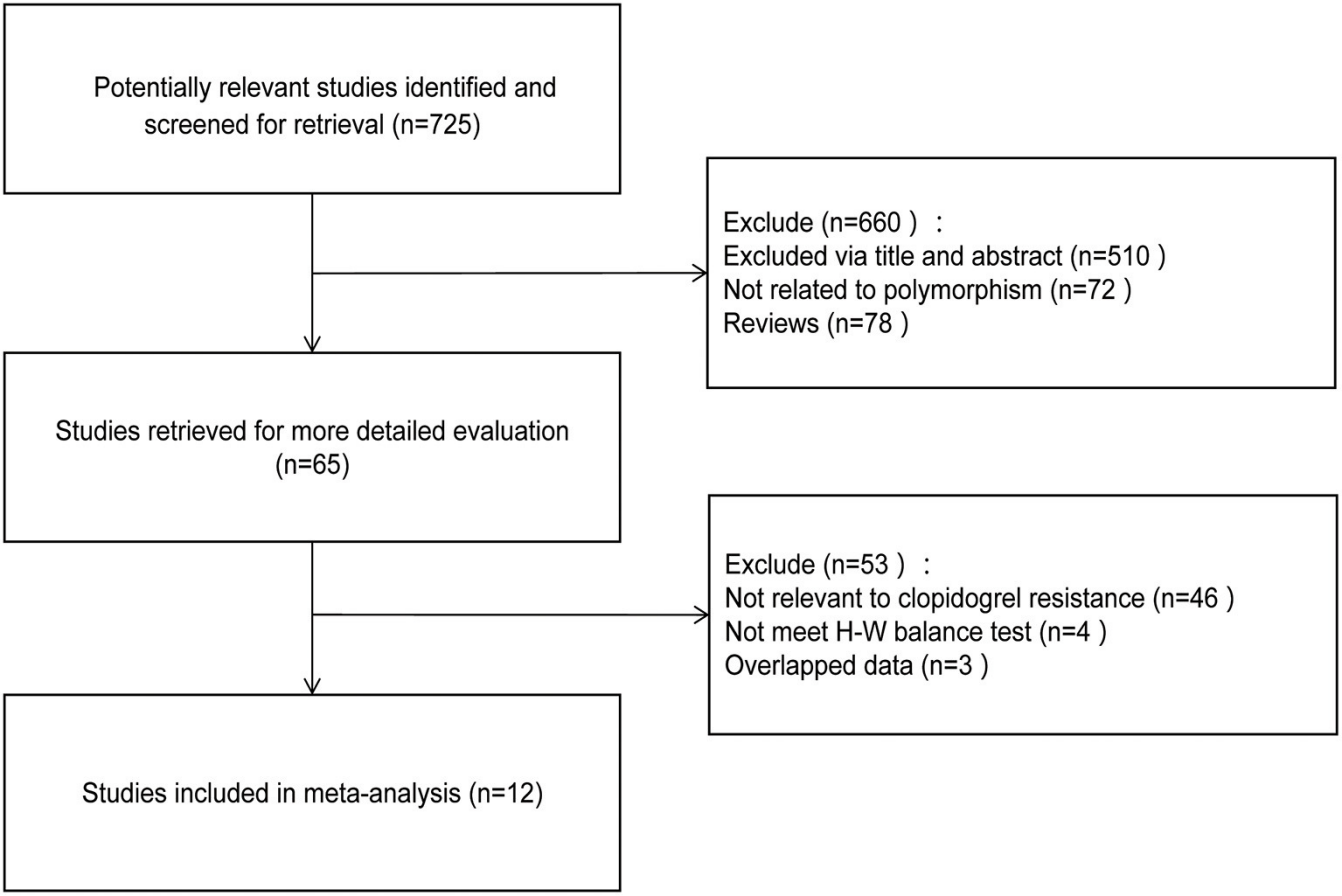
A flow diagram of the study selection process.

A total of 12 trials were selected according to the criteria (Chen et al., 2010; Cuisset et al., 2011; Hwang et al., 2011; Li et al., 2013, 2018; Zhang et al., 2013; Amin et al., 2017; Liang et al., 2017; Saydam et al., 2017; Shen et al., 2017; Wang et al., 2018; Zhuo et al., 2018), including 10 from Asian population and 2 from Caucasian population. A total of 3,073 subjects were involved, including 1,174 patients with clopidogrel resistance and 1,899 patients with non-clopidogrel resistance. The specific screening process can be found in Figure 1. The characteristics of each study and the genotype distribution reported in the study can be found in Table 1. The results of NOS quality evaluation of the literature were shown in Supplementary Table 2. Thus, it can be seen that the NOS scores of the studies included in this study were all above 6, which belonged to high-quality research.

**Table 1:** Characters of included studies.

**References**	**Mean age**	**Country**	**Sample (CR/NCR)**	**Definition of CR**	**CLD (mg)**	**CR**			**NCR**			**HWE**	**NOS score**
						**AA**	**GA**	**GG**	**AA**	**GA**	**GG**		
Chen et al. (2010)	67.5	China	187/228	Percent inhibition of ADP ≤ 10%	300	17	98	72	8	93	127	0.0672	7
Cuisset et al. (2011)	64	France	151/195	VASP index > 50%	600	11	35	105	2	28	165	0.5135	8
Hwang et al. (2011)	62.6	Korea	91/99	ADP-induced MPA > 50%	300	13	44	34	5	35	59	0.9481	8
Zhang et al. (2013)	63.9	China	164/336	ADP-induced MPA > 50%	300	24	77	63	21	139	176	0.3494	8
Li et al. (2013)	59.6 ± 6.5	China	127/335	Percent inhibition of ADP ≤ 10%	600	31	57	39	27	134	174	0.8659	7
Shen et al. (2017)	63.69 ± 11.04	China	78/151	ADP-induced MPA > 50% or Percent inhibition of ADP ≤ 10%	NR	10	42	26	2	51	98	0.1003	7
Liang et al. (2017)	61.22 ± 10.31	China	70/91	ADP-induced MPA > 50%	300	14	44	12	3	37	51	0.2270	7
Saydam et al. (2017)	62 (54–70)	Turkey	104/243	PRU > 208	75	7	40	57	5	42	196	0.1364	8
Amin et al. (2017)	NR	Malaysia	27/44	PRU > 208	600	8	8	11	3	22	19	0.3121	8
Zhuo et al. (2018)	69.71 ± 13.44	China	26/65	IPA <30%	300	4	15	7	3	31	31	0.1676	8
Wang et al. (2018)	61 ± 10	China	137/104	PRI ≤ 50%	75	1	38	65	11	66	60	0.2198	8
Li et al. (2018)	63.5 ± 11.0	China	34/162	IPA < 30%	75	9	19	6	6	33	123	0.0589	8

*CR, clopidogrel resistance; NCR, non-clopidogrel resistance; CLD, clopidogrel loading dose; HWE, Hardy-Weinberg Equilibrium; ADP, adenosine diphosphate; VASP, vasodilator phosphoprotein; MPA, maximal platelet aggregation; IPA, inhibition of platelet aggregation; PRI, platelet reaction index; PRU, P2Y12 reaction unit; NOS, Newcastle-Ottawa quality assessment scale*.

### Meta-Analysis Results

#### Allele Contrast

**Figure 2:**
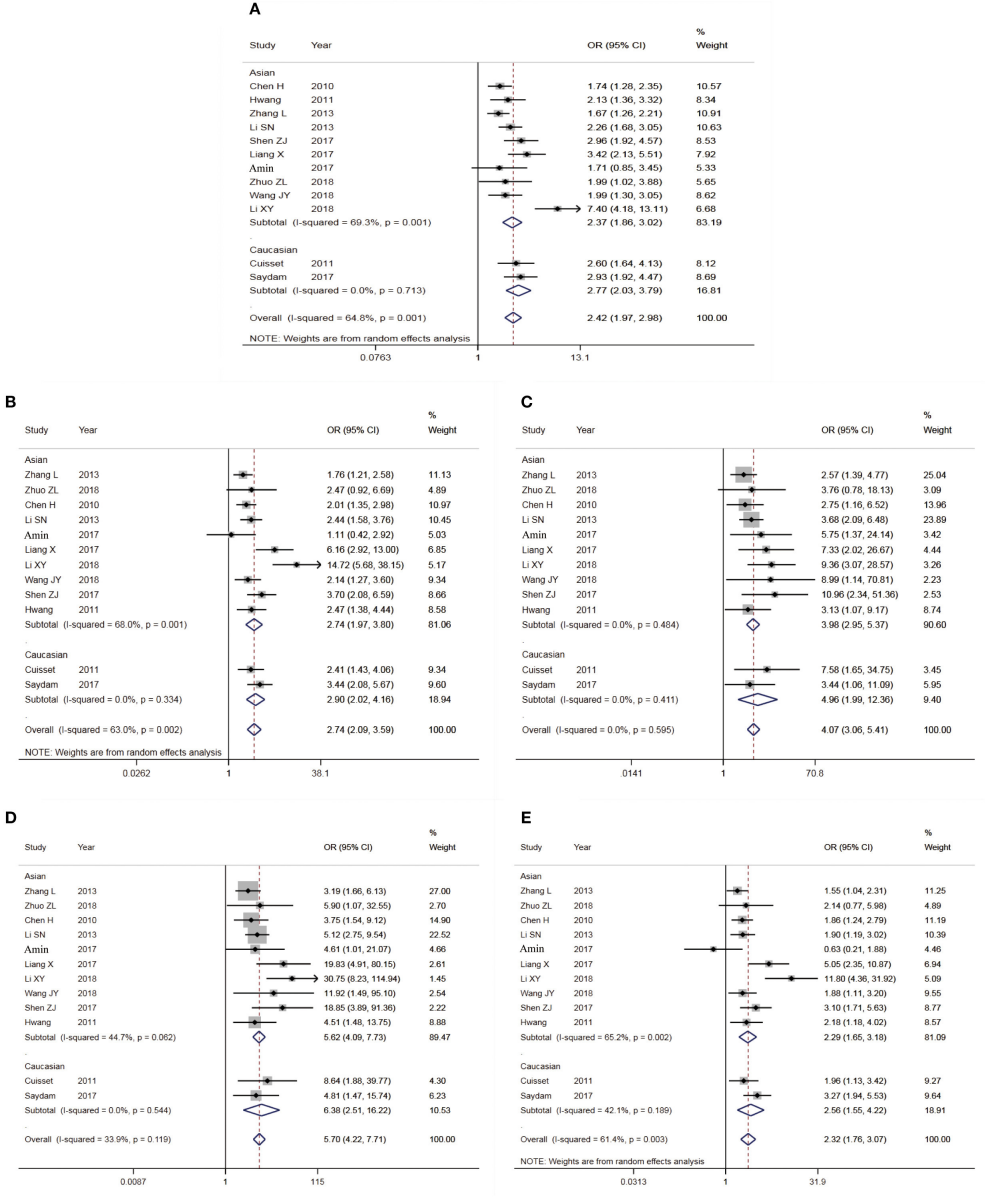
Forest plot for the association between cyp2c19*2 Polymorphism and Clopidogrel resistance (**A**: Allelic model; **B**: Dominant model; **C**: Recessive model; **D**: Homozygous model; **E**: Heterozygote model).

**Figure 3:**
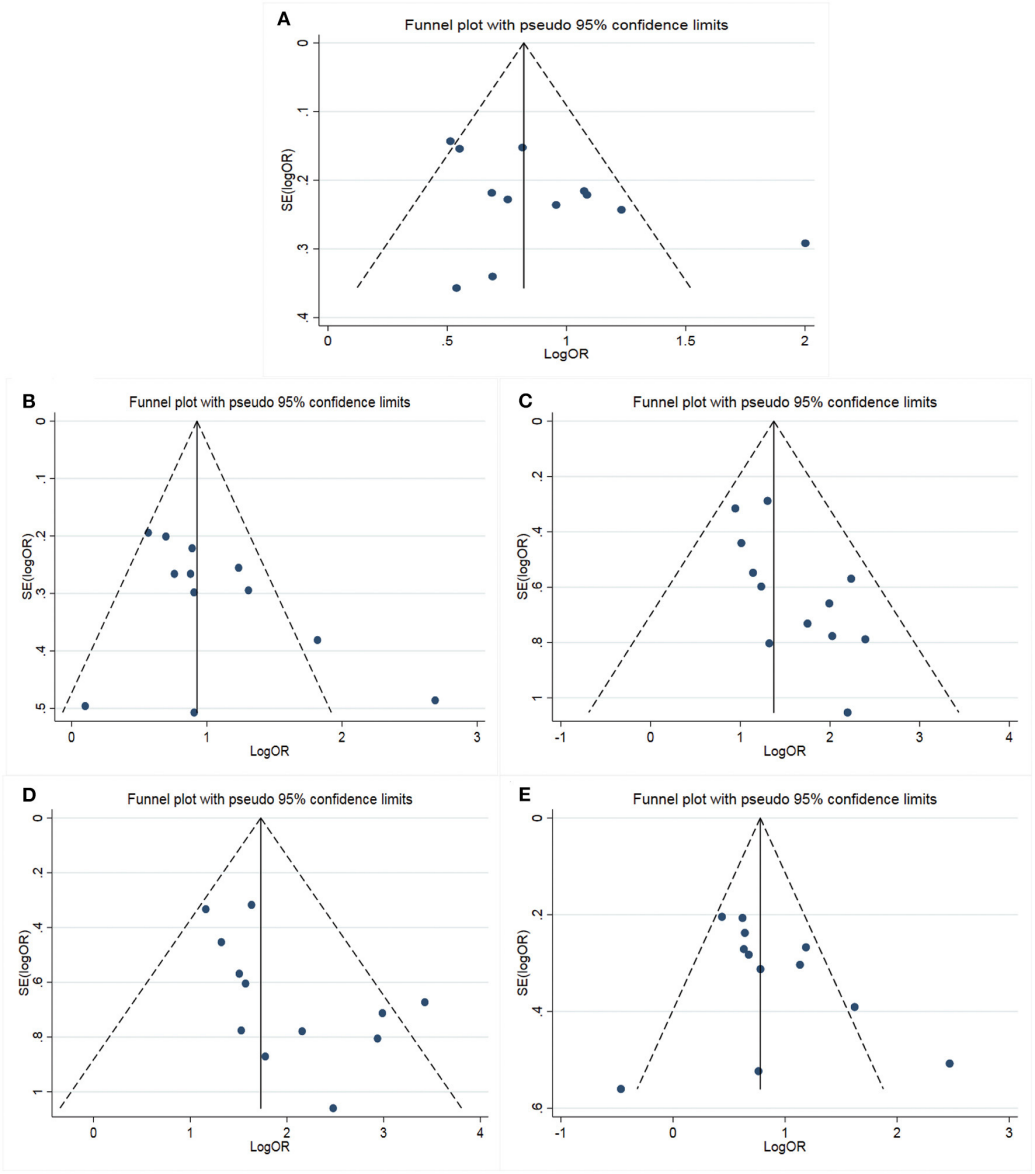
Funnel plot for the assessment of publication bias (**A**: Allelic model; **B**: Dominant model; **C**: Recessive model; **D**: Homozygous model; **E**: Heterozygote model).

The main results of meta-analysis were shown in Table 2 and Figure 2. Compared A allele with G allele, I^2^ = 64.8%, *P* < 0.05, which indicated that the heterogeneity among studies was statistically significant, and random effect model was used. The final results showed that OR = 2.42 (95%CI: 1.97–2.98, *P* < 0.01), the difference was statistically significant. According to the Ethnicity subgroup analysis, the results showed that there was a significant difference in Asian population with OR = 2.37 (95%CI: 1.86–3.02, *P* < 0.01), and there was also statistical significance in Caucasian population shown in Figure 2A. This suggested that there was a correlation between Cyp2C19*2 polymorphism and clopidogrel resistance. The funnel plot in Figure 3A was basically symmetrical, and Egger's Test showed that *P*-value was >0.05, which indicated that there was no publication bias.

**Table 2:** Results of meta-analysis for cyp2c19^*^2 Polymorphism and Clopidogrel resistance.

**Genetic models**	**Subgroup**	***n***	**OR**	**95%CI**	***P***	***I*^**2**^ (%)**	***P* for heterogeneity**	**Model**	***P* for Publication bias**
Allelic model (A vs. G)	Overall	12	2.42	1.97–2.98	0.000	64.8	0.001	REM	0.125
Caucasian	Asian	10	2.37	1.86–3.02	0.000	69.3	0.001	REM	0.178
	Caucasian	2	2.77	2.03–3.79	0.000	0.0	0.731	FEM	NA
Year	≤ 2013	5	1.96	1.68–2.30	0.000	7.2	0.366	FEM	0.223
	>2013	7	2.88	2.07–3.99	0.000	65.1	0.000	REM	0.795
Definition of clopidogrel resistance	Percent inhibition of ADP ≤ 10%	2	1.98	1.53–2.57	0.000	32.4	0.224	REM	NA
	ADP-induced MPA > 50%	3	2.22	1.47–3.36	0.000	69.3	0.038	REM	0.363
	Others	7	2.76	2.00–3.80	0.000	64.0	0.011	REM	0.766
Dominant model (AA + AG vs. GG)	Overall	12	2.74	2.09–3.59	0.000	63.0	0.002	REM	0.063
Caucasian	Asian	10	2.74	1.97–3.80	0.000	68.0	0.001	REM	0.093
	Caucasian	2	2.90	2.02–4.16	0.000	0.0	0.334	FEM	NA
Year	≤ 2013	5	2.12	1.74–2.59	0.000	0.0	0.760	FEM	0.116
	>2013	7	3.49	2.15–5.69	0.000	70.3	0.003	REM	0.361
Definition of clopidogrel resistance	Percent inhibition of ADP ≤ 10%	2	2.19	1.64–2.93	0.000	0.0	0.516	FEM	NA
	ADP-induced MPA > 50%	3	2.81	1.44–5.50	0.002	76.8	0.013	REM	0.296
	Others	7	3.03	1.96–4.68	0.000	66.6	0.006	REM	0.361
Recessive model (AA vs. AG + GG)	Overall	12	4.07	3.06–5.41	0.000	0.0	0.595	FEM	0.017
Caucasian	Asian	10	3.98	2.95–5.37	0.000	0.0	0.484	FEM	0.031
	Caucasian	2	4.96	1.99–12.36	0.001	0.0	0.411	FEM	NA
Year	≤ 2013	5	3.25	2.31–4.59	0.000	0.0	0.721	FEM	0.306
	>2013	7	6.52	3.86–11.01	0.000	0.0	0.856	FEM	0.778
Definition of clopidogrel resistance	Percent inhibition of ADP ≤ 10%	2	3.34	2.07–5.38	0.000	0.0	0.578	FEM	NA
	ADP-induced MPA > 50%	3	3.25	2.00–5.29	0.000	4.0	0.353	FEM	0.386
	Others	7	6.53	3.81–11.17	0.000	0.0	0.855	FEM	0.740
Homozygous model(AA vs. GG)	Overall	12	5.70	4.22–7.71	0.000	33.9	0.119	FEM	0.085
Caucasian	Asian	10	5.62	4.09–7.73	0.000	44.7	0.062	FEM	0.097
	Caucasian	2	6.38	2.51–16.22	0.000	0.0	0.544	FEM	NA
Year	≤ 2013	5	4.31	2.99–6.21	0.000	0.0	0.734	FEM	0.320
	>2013	7	10.52	6.01–18.41	0.000	15.7	0.310	FEM	0.832
Definition of clopidogrel resistance	Percent inhibition of ADP ≤ 10%	2	4.58	2.74–7.64	0.000	0.0	0.572	FEM	NA
	ADP-induced MPA > 50%	3	4.62	2.79–7.67	0.000	63.1	0.067	REM	0.392
	Others	7	9.18	5.18–16.26	0.000	4.5	0.393	FEM	0.854
Heterozygous model (AG vs. GG)	Overall	12	2.32	1.76–3.07	0.000	61.4	0.003	REM	0.085
Caucasian	Asian	10	2.29	1.65–3.18	0.000	65.2	0.002	REM	0.113
	Caucasian	2	2.56	1.55–4.22	0.000	42.1	0.189	FEM	NA
Year	≤ 2013	5	1.82	1.48–2.25	0.000	0.0	0.897	FEM	0.095
	>2013	7	2.91	1.75–4.85	0.000	70.4	0.002	REM	0.443
Definition of clopidogrel resistance	Percent inhibition of ADP ≤ 10%	2	1.88	1.38–2.55	0.000	0.0	0.947	FEM	NA
	ADP-induced MPA > 50%	3	2.41	1.27–4.57	0.007	72.5	0.026	REM	0.294
	Others	7	2.52	1.58–4.03	0.000	68.2	0.004	REM	0.439

*OR, odds ratio; FEM, fixed-effects model; REM, random-effects model*.

#### Dominant Genetic Model

The genotype AA + GA vs. the genotype GG, I^2^ = 63.0%, *P* < 0.05, which indicated that the heterogeneity among the studies was statistically significant, and the random effect model was used. The results showed that there was a significant difference with OR = 2.74 (95%CI: 2.09–3.59, *P* < 0.01). The ethnic subgroup analysis showed the same results, the comparison of Caucasian and Asian dominant genetic models was statistically significant in Figure 2B. The funnel plot was basically symmetrical shown in Figure 3B, Egger's Test suggested that *P*-value was >0.05, which meant that the publication bias was well-controlled.

#### Recessive Genetic Model

The genotype AA was compared with genotype GA + GG, I^2^ = 0.0%, *P* > 0.05, which suggested that there was no statistical significance in the heterogeneity among the studies, and the fixed effect model was used. The results showed that there was a significant difference with OR = 4.07 (95%CI: 3.06–5.41, *P* > 0.05). The ethnic subgroup analysis indicated the same results, the comparison of Caucasian and Asian recessive genetic models was statistically significant, and was shown in Figure 2C. The symmetry of funnel plot showed bias (Figure 3C), Egger's Test showed *P* < 0.05, which indicated that there was a certain bias.

#### Homozygous Model

The genotype AA vs. GG, I^2^ = 33.9%, *P* > 0.05, which showed that there was no statistical significance in the heterogeneity among the studies, and the fixed effect model was used. The final results showed that there was a significant difference with OR = 5.70 (95%CI: 4.22–7.71, *P* < 0.01). The ethnic subgroup analysis indicated the same results, the comparison of Caucasian and Asian homozygous genetic models was statistically significant shown in Figure 2D. The funnel plot was basically symmetrical (Figure 3D), Egger's Test showed that *P* > 0.05, which suggested that the publication bias was well-controlled.

#### Heterozygote Model

The genotype GA compared with GG, I^2^ = 61.4%, *P* < 0.05, which showed that the heterogeneity among the studies was statistically significant, and the random effect model was used. The final results showed that there was a significant difference with OR = 2.32 (95%CI: 1.76–3.07, *P* > 0.05). The ethnic subgroup analysis suggested the same results, the comparison of Caucasian and Asian heterozygous genetic models was statistically significant, and the forest plot was shown in Figure 2E. The funnel plot was basically symmetrical (Figure 3E), Egger's Test showed that *P* > 0.05, the difference was not statistically significant, which indicated that the publication bias was well-controlled.

### Subgroup Analysis of Year and CR Definition

The results of subgroup analysis of the published year (Table 2) showed that the differences were statistically significant in all genetic models. Moreover, the heterogeneity of studies before 2013 decreased significantly.

The results of subgroup analysis defined by CR (Table 2) showed that the differences were statistically significant in all genetic models. Moreover, when CR was defined as Percent inhibition of ADP ≤ 10%, the heterogeneity of the study decreased significantly.

### Sensitivity Analysis

The results of the sensitivity analysis were shown in Supplementary Figures 1A–E. Each study was excluded one by one and meta-analysis was used. The results of the meta-analysis showed that the combined effect of the allele model and the other four gene models did not change significantly after the removal of a single study, indicating that the results were robust.

## Discussion

The polymorphism of *Cyp2C19* gene determines the difference of its enzymes among individuals, which affects the concentration of the metabolically active products and anti-platelet aggregation of clopidogrel (Watala et al., 2004). Brandt et al. (2007) found that the level of active metabolites of clopidogrel in people with defective Cyp2C19*2 gene was significantly lower than that in non-carriers after taking clopidogrel. Geisler et al. (2008) found that after the first administration of loading dose of clopidogrel, the degree of platelet aggregation induced by ADP was closely related to *Cyp2C19* genotype, and the residual platelet aggregation (RPA) was significantly increased in patients with Cyp2C19*2 genotype. Pettersen et al. (2011) measured the platelet function of patients taking clopidogrel. By measuring *P2Y12-PRU* and VASP-PRI, it was observed that Cyp2C19*2 carriers had higher platelet activity than non-carriers. Lee et al. (2011) also obtained the same conclusion by testing *P2Y12-PRU* in his study. Previous studies have different definition of clopidogrel resistance, and there are no recognized indicators at home and abroad to define clopidogrel resistance. Currently, the definition of clopidogrel resistance is that the ADP-induced decrease in maximal platelet aggregation rate is <10% compared with baseline (Chen et al., 2010). Clopidogrel resistance used by Ma et al. (2010) was defined by flow cytometry with the platelet reactivity index of vasodilator-stimulated phosphoprotein (VASP-PRI), VASP-PRI >50%. Parodi et al. (2011) detected ADP-induced platelet aggregation in all patients, defined platelet aggregation rate >70% as high residual platelet reactivity (HRPR), platelet aggregation rate <70% as low residual platelet reactivity (LRPR), followed up for 2 years, compared with the LRPR group, the HRPR group had a higher incidence of ST and major end point events. In this study, multiple studies on the definition of clopidogrel resistance were integrated, and domestic and foreign studies on the correlation between Cyp2C19*2 gene polymorphism and clopidogrel resistance in coronary heart disease were collected to comprehensively analyze the relationship between them, so as to provide more in-depth evidence-based medicine for clinical practice.

According to the strict inclusion and exclusion criteria, this study included 12 high-quality literatures with a total of 3,073 subjects. The results showed that the allele model (A vs. G): OR = 2.42 (95%CI: 1.97–2.98), the difference was statistically significant. Dominant gene model, recessive gene model, homozygote model, and heterozygote model were also statistically significant. The funnel chart and Egger's Test results of publication bias showed that there was no publication bias. The results of subgroup analysis showed that the results of the Asian population were consistent with those of the total population, while the Caucasian population was inconclusive because only two articles were included. The results of subgroup analysis of different definitions of clopidogrel resistance and the year of publication showed that the differences were statistically significant in each genetic model of each subgroup. Sensitivity analysis results indicated that after excluding a single study, the meta-analysis results of the allele model and the other four gene models did not have a statistically significant change, indicating that the results were robust. Therefore, it could be considered that there is a strong association between Cyp2P19*2 polymorphism and clopidogrel resistance in patients with CHD, and the variant of allele G could increase clopidogrel resistance in antiplatelet therapy. This is basically consistent with the conclusion of a previous meta-analysis by Hou et al. (2014) including eight studies. Furthermore, compared with previous studies, this study is more stringent in the literature inclusion criteria, such as excluding the study that the control group does not conform to HWE, and including more latest studies. From the publication bias and stability results, the conclusions of this study are reliable and valuable.

In this study, several limitations should be noted: (1) Since there is no standard definition of clopidogrel resistance at present, multiple definitions of clopidogrel resistance were used in this study, which may weaken the comparability of the data; (2) In the subgroup analysis, the sample size is relatively small, and there are only 2 studies in the Caucasian population, so the conclusion need to be carefully adapted to the Caucasian population; (3) The lack of raw data also limits further assessment of potential gene-gene or gene-environment interactions.

In conclusion, Cyp2C19*2 gene polymorphism is associated with clopidogrel resistance reflected by platelet function assay, and the allele (A vs. G) increases clopidogrel resistance, which is reflected in the other four models, especially in the Asian population. This conclusion can be used to guide the individualized antiplatelet therapy of clopidogrel. Due to the limitations of this study, such as multiple definitions of clopidogrel resistance, gene-gene interaction, it is necessary to do more and more in-depth studies on Cyp2C19*2 gene polymorphism and antiplatelet therapy.

## Author Contributions

YS, GY, and QL have given substantial contributions to the conception or the design of the manuscript, GY and YS to acquisition, analysis, and interpretation of the data. All authors have participated to drafting the manuscript, BC and GY revised it critically. All authors read and approved the final version of the manuscript.

## Conflict of Interest

The authors declare that the research was conducted in the absence of any commercial or financial relationships that could be construed as a potential conflict of interest.

## Supplementary Material

Supplementary Figure 1 Sensitivity analysis results (**A**: Allelic model; **B**: Dominant model; **C**: Recessive model; **D**: Homozygous model; **E**: Heterozygote model).

Supplementary Table 1 PRISMA checklist.

Supplementary Table 2 Quality assessment of the studies included in this meta-analysis (NOS).

## Abbreviations

CAHD: coronary atherosclerotic heart disease
CHD: coronary heart disease
CNKI: National Knowledge Infrastructure
CR: clopidogrel resistance
EMBASE: Excerpt Medica Database
FEM: fixed-effects model
HRPR: high residual platelet reactivity
HWE: Hardy-Weinberg equilibrium
LRPR: low residual platelet reactivity
NOS: Newcastle-Ottawa Scale
PCI: percutaneous coronary intervention
REM: random-effects model
ST: stent thrombosis
RPA: residual platelet aggregation
VASP-PRI: vasodilator-stimulated phosphoprotein.
