# Supplementary figures and images for: Cyp2C19*2 Polymorphism Related to Clopidogrel Resistance in Patients With Coronary Heart Disease, Especially in the Asian Population: A Systematic Review and Meta-Analysis
Source: Front Genet. 2020 Dec 22;11:576046. doi: 10.3389/fgene.2020.576046 (PMC7783419; doi:10.3389/fgene.2020.576046)

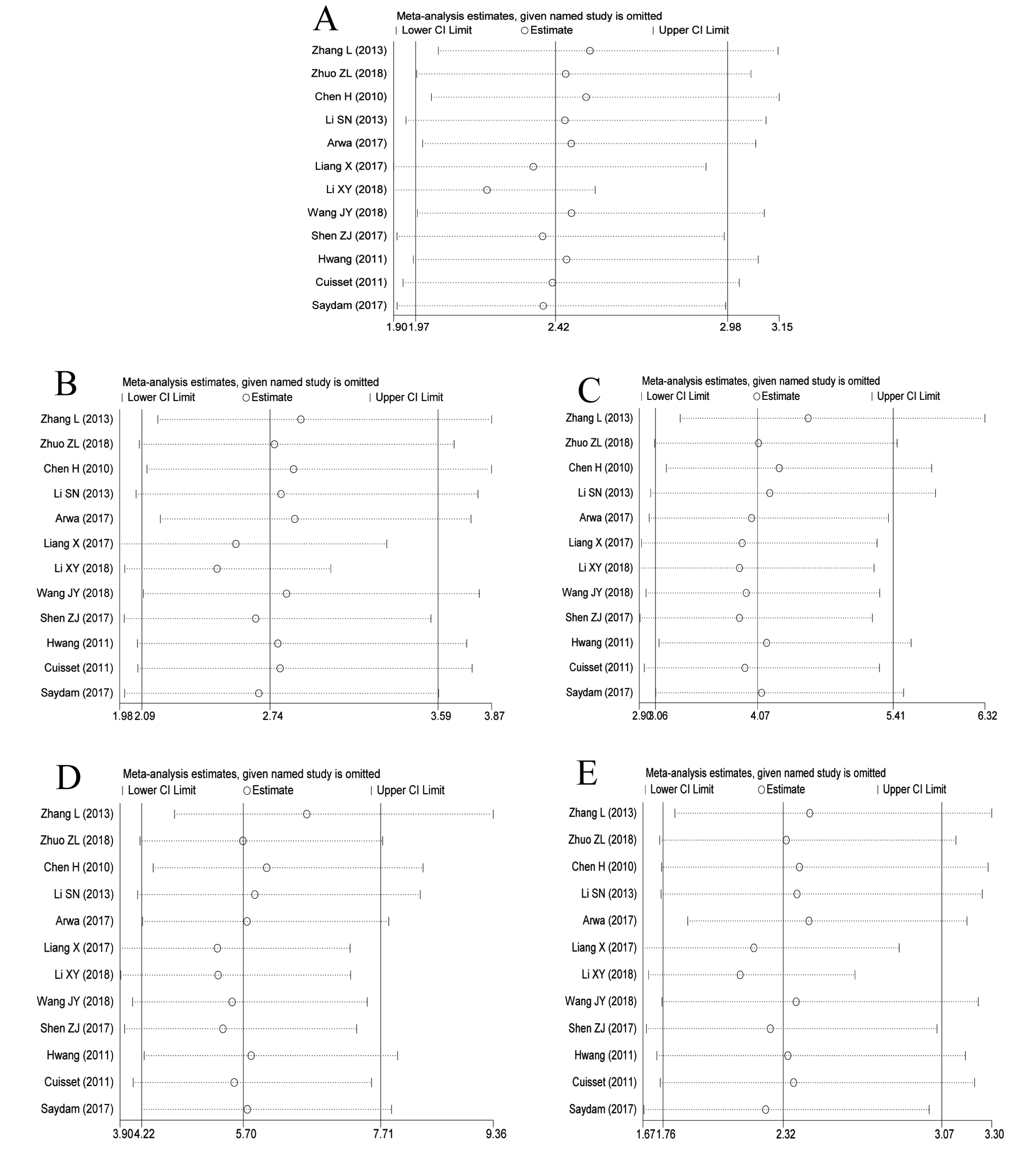

Supplement: Supplementary Figure 1 — Sensitivity analysis results (A: Allelic model; B: Dominant model; C: Recessive model; D: Homozygous model; E: Heterozygote model). [file Image_1.tif]
