# Supplementary material for: Cyp2C19*2 Polymorphism Related to Clopidogrel Resistance in Patients With Coronary Heart Disease, Especially in the Asian Population: A Systematic Review and Meta-Analysis
Source: Front Genet. 2020 Dec 22;11:576046. doi: 10.3389/fgene.2020.576046 (PMC7783419; doi:10.3389/fgene.2020.576046)
Supplement: Supplementary Table 2 — Quality assessment of the studies included in this meta-analysis (NOS). [file Table_2.doc]

Supplementary table 2 Quality assessment of the studies included in this meta-analysis(NOS)

| First author | Year | Case selection | | | |  | Comparability between groups | |  | Exposure factor measurement | | | NOS score |
| --- | --- | --- | --- | --- | --- | --- | --- | --- | --- | --- | --- | --- | --- |
|  |  | 1 | 2 | 3 | 4 |  | 5 | 6 |  | Blind method | 7 | Response rate |  |
| Chen et al | 2010 | * | * | * | * |  | * | ? |  | ? | * | * | 7 |
| Cuisset et al | 2011 | * | * | * | * |  | * | * |  | ? | * | * | 8 |
| Hwang et al | 2011 | * | * | * | * |  | * | * |  | ? | * | * | 8 |
| Zhang L et al | 2013 | * | * | * | * |  | * | * |  | ? | * | * | 8 |
| Li SN et al | 2013 | * | * | * | * |  | * | ? |  | ? | * | * | 7 |
| Shen ZJ et al | 2017 | * | * | * | * |  | * | * |  | ? | ? | * | 7 |
| Liang X et al | 2017 | * | * | * | * |  | * | ? |  | ? | * | * | 7 |
| Saydam et al | 2017 | * | * | * | * |  | * | * |  | ? | * | * | 8 |
| Arwa et al | 2017 | * | * | * | * |  | * | * |  | ? | * | * | 8 |
| Zhuo ZL et al | 2018 | * | * | * | * |  | * | * |  | ? | * | * | 8 |
| Wang JY et al | 2018 | * | * | * | * |  | * | * |  | ? | * | * | 8 |
| Li XY et al | 2018 | * | * | * | * |  | * | * |  | ? | * | * | 8 |

Note:*:Yes;?:unclear;1:Case identification appropriate;2:Case representativeness;3:The source of the control clear;4:control group choosed properly;5:Controls the most important confounding factors;6:Control other confounding factors;7:same exposure determination method; NOS:Newcastle-Ottawa Scale
